# Patients’ and carers’ experiences of interacting with home haemodialysis technology: implications for quality and safety

**DOI:** 10.1186/1471-2369-15-195

**Published:** 2014-12-11

**Authors:** Atish Rajkomar, Ken Farrington, Astrid Mayer, Diane Walker, Ann Blandford

**Affiliations:** UCL Interaction Centre, University College London, London, UK; Renal Medicine, East and North Hertfordshire NHS Trust, Stevenage, UK; UCL Medical School, University College London, London, UK; Renal Unit, Royal Free Hospital, London, UK

**Keywords:** Home haemodialysis, Human factors, Medical device design, Patient safety, Patient satisfaction, User-computer interface

## Abstract

**Background:**

Little is known about patients’ and carers’ experiences of interacting with home haemodialysis (HHD) technology, in terms of user experience, how the design of the technology supports safety and fits with home use, and how the broader context of service provision impacts on patients’ use of the technology.

**Methods:**

Data were gathered through ethnographic observations and interviews with 19 patients and their carers associated with four different hospitals in the UK, using five different HHD machines. All patients were managing their condition successfully on HHD. Data were analysed qualitatively, focusing on themes of how individuals used the machines and how they managed their own safety.

**Results:**

Findings are organised by three themes: learning to use the technology, usability of the technology, and managing safety during dialysis. Home patients want to live their lives fully, and value the freedom and autonomy that HHD gives them; they adapt use of the technology to their lives and their home context. They also consider the machines to be safe; nevertheless, most participants reported feeling scared and having to learn through mistakes in the early months of dialysing at home. Home care nurses and technicians provide invaluable support. Although participants reported on strategies for anticipating problems and keeping safe, perceived limitations of the technology and of the broader system of care led some to trade off safety against immediate quality of life.

**Conclusions:**

Enhancing the quality and safety of the patient experience in HHD involves designing technology and the broader system of care to take account of how individuals manage their dialysis in the home. Possible design improvements to enhance the quality and safety of the patient experience include features to help patients manage their dialysis (e.g. providing timely reminders of next steps) and features to support communication between families and professionals (e.g. through remote monitoring).

## Background

While most haemodialysis take place within dialysis centres (in-centre HD), treatment may also take place in the patient’s home (HHD); it is argued that home treatment is substantially cheaper, at least in the long term [1], and also the modality of choice for many patients [2, 3]. Professionals have been found to favour HHD over in-centre HD, particularly for intensive treatment regimes [4, 5]. However, HHD treatment is complex, requiring many steps to be performed in the correct order, and fatal incidents involving HHD machines have been reported [6]. For example, Allcock et al. [7] report on a fatal incident in which the patient connected the arterial line instead of the venous line to the saline bag, resulting in exsanguination. Morton et al. [8] present a systematic review of qualitative studies of patient care in chronic kidney disease; of the 18 studies included, most concerned patients’ treatment preferences; three focused on experiences of transplantation and one on palliative care. No studies to date have focused on evaluating the user-machine interaction design of HHD machines. This study aimed to understand the issues that patients and carers face when interacting with HHD machines, to identify safety-related implications and user-centred requirements for next-generation HHD technology. Through the study, it also became apparent that features of the broader context of care influences patients’ use and experience of the technology.

Although some studies of professionals’ attitudes [4, 5] focus principally on patient outcomes, a US study [9] found that the main concern that nephrologists have when motivating patients to go on HHD was patient safety. An Australian study of patients and their carers [10] also found that patients value survival and convenience in choosing treatment, a finding echoed by Cafazzo et al. [11] in their study of nocturnal HHD. A study conducted in Italy found that patients and carers believe that performing HHD means abandoning the sense of security provided by a supervised dialysis unit [12]. Rygh et al. [13] interviewed eleven patients on home dialysis (three of whom were on HHD) in Norway; they focus on how people learn to cope, emphasising quality of life and the importance of very close contact with hospital staff. Enhancing the quality and safety of the home patient experience through the design of HHD technology and the broader system of care could encourage more patients to shift from in-centre haemodialysis to HHD, which is a current goal in the UK and other countries.

### Qualitative research in healthcare: an overview

The study reported here uses a qualitative methodology because this suited the research questions being addressed, which focus on patients’ experiences and practices.

Much has been written about the role, status and value of qualitative research in healthcare. Shuval et al. [14] tracked the growth of qualitative studies being published in medical journals over a decade (1997–2007), noting that they represented 4.1% of all research papers in 2007 (compared with 1.2% in 1998) across the 67 journals included in their study.

Qualitative research is widely regarded as “unscientific and anecdotal” [15] and based on evidence of a “lower order” [16] than that of randomised controlled trials. Rather than being superior or inferior, many researchers [14–18] argue that qualitative and quantitative research methods are better regarded as complementary: each suited to addressing particular kinds of research questions. Green and Britten [15] note that “The value of qualitative methods lies in their ability to pursue systematically the kinds of research questions that are not easily answerable by experimental methods”. They argue that qualitative research is marked by a different orientation to research compared with quantitative methods, including a focus on understanding treatment within its every-day context. Similarly, Shuval et al. [14] note that “Qualitative research enables determining ‘how’ and ‘why’ evidence is translated into clinical practice, for example, in comparison to ‘what’ evidence is translated into practice as derived from quantitative research”. Whilst qualitative research can be confused with anecdotes and opinions, this overlooks the systematic and critical approach applied in rigorous qualitative research based on explicit sampling strategies, systematic analysis of data and a commitment to examining counter explanations [15, 16].

There is an extensive literature on what constitutes quality in qualitative research [19, 20]. Malterud [18] notes that whilst the criteria that are traditionally applied to quantitative studies (internal and external validity, objectivity, generalisability, etc.) do not readily translate to qualitative studies, there are three key standards for qualitative studies: relevance, validity and reflexivity. We have conducted this study according to these standards.

## Methods

Ethical clearance was obtained from a UK National Health Service Research Ethics Committee (reference 11/LO/0329). An availability sampling approach was used: one patient responded to an open letter sent to the National Kidney Federation website; all other patients were invited to participate in the study by hospital staff. All were patients who were perceived by staff as managing their own care well. All those who expressed an interest subsequently gave informed consent and participated. Data were gathered through ethnographic observations and interviews with 19 patients; carers also participated in nine of the interviews. The patients were attached to four different hospitals in the UK. Additionally, three home nurses, three renal technicians, and one nephrologist were interviewed.

Each patient was observed during a dialysis session, and then interviewed on how they carried out their treatment. The extent of the observation varied, depending on what each participant was comfortable with, and on average lasted an hour. In some cases the period of observation covered the patient’s preparation for dialysis, the setting up of the machine, and the initial part of the treatment, while in other cases the observation covered the last part of the treatment, disconnection and clearing up. Each interview was semi-structured and focused on eliciting incidents and near misses that patients and carers had experienced, the issues they faced when interacting with HHD technology, and their strategies for coping with these issues. Data were collected in the form of field notes, audio-recorded interviews and, with the participant’s permission, photographs of the physical environment in which they dialysed and of artefacts used. All interviews were transcribed for subsequent analysis.

The observation notes, interview transcripts, pictures and interview notes were coded systematically to identify patients’ strategies and issues when interacting with HHD technology. Thematic analysis [21] was applied to draw out three inter-related aspects of patients’ and carers’ experiences, presented in the next section. Rather than being a full ‘bottom up’ analysis, this analysis started from an interest in how patients and their families experienced HHD technology and how their safety might be affected by their use of technology. We took a critical stance: for example, seeking out aspects of design that enhanced safety as well as those that might compromise it, and aspects that participants found easy to use as well as those that presented difficulties. Where different participants reported experiences that appeared to contradict each other, we probed deeper to understand the sources of the differences.

As a further stage of analysis (presented as Discussion below), we reviewed the findings to identify requirements for both future HHD technology and the broader system of care.

Little quantitative analysis is presented because too much variability was found to present meaningful quantitative results. For example, while ten participants in the study had a carer who had been trained in some aspects of HHD procedures, only five carers routinely took the lead in managing the HHD procedure and yet others relied on a carer (family member or neighbour) to perform significant parts of the procedure, or to intervene in case of emergency, and one of the fully trained carers delegated part of the set-up procedure to another family member, so it is not informative to report a definitive number of carers.

## Results

Patients had varying backgrounds: age ranged from 24 to 77 and HHD vintage from 3 weeks to 30 years. Between them, they were using 5 different haemodialysis machines from leading international manufacturers. Participant profiles are summarised in Table 1.

**Table 1:** **Participant profiles**

Patient	Gender	Age	Carer	Helper	Other conditions	On dialysis	On HHD	Hospital	Machine
P1	M	30+		Wife	Diabetes	3 yrs	4 wks	H1	M1
P2	M	70+	Son	Wife	Heart disease	1 yr	3 wks	H1	M1
P3	M	70+	Wife		Paraplegic, diabetes	2.5 yrs	3 mths	H1	M1
P4	F	20+				13 yrs	1.5 yrs	H1	M2
P5	F	60+				15 yrs	10 yrs	H1	M2
P6	M	70+		Wife	Has pacemaker	8 yrs	3 wks	H1	M1
P7	F	40+		Mother	Arthritis	27 yrs	10 yrs	H1	M2
P8	F	30+		Partner, daughter		17 yrs	1.5 yrs	H2	M3
P9	M	70+	Wife		Heart problems +	2.5 yrs	2 yrs	H3	M4
P10	F	60+	Husband			4.5 yrs	3 yrs	H3	M4
P11	F	60+	Husband		Diabetes	n/a	9 mths	H3	M5
P12	M	50+		Wife		3 yrs	1.5 yrs	H3	M4
P13	M	40+	Wife		Hernia	2.5 yrs	1.5 yrs	H3	M3
P14	F	50+	Husband			1 yr	1 yr	H3	M3
P15	M	60+	Wife			4 yrs	2 yrs	H3	M5
P16	M	20+		Mother		3 yrs	2 yrs	H3	M3
P17	F	40+		Mother	Impaired vision	18 yrs	8.5 yrs	H4	M5
P18	F	60+	Husband		Prosthetic leg	35 yrs	30 yrs	H4	M5
P19	F	60+	Son	Daughter-in-law	Diabetes, impaired vision	6 yrs	1 mt	H4	M5

Sessional frequency varied from thrice weekly to daily (none were on nocturnal HHD); employment status from retired to full-time employed; family situation from living alone to living with spouse and children; all managed their own care without nurse-assisance.

As noted above, our focus is on how people use HHD technology and implications for patient experience and safety; within this area of interest, three main themes were the focus of the analysis: learning to use the technology, usability of the technology, and managing safety during dialysis. These themes are interdependent: in the early stages of HHD, the challenges of learning to use the technology and to manage one’s own dialysis affect both safety and user experience, and the usability of the machine is one important factor contributing to ease of learning. Throughout, features of the broader system of care, such as the support provided by key clinicians and technicians or anticipated difficulties in getting a place for in-centre HHD at short notice, also influence patients’ and carers’ experiences, actions and safety. The key themes and findings are summarised in Table 2 and discussed below.

**Table 2:** **Summary of findings on learning, usability and managing safety**

Broad theme	Aspect	Details
Learning to use technology	Early experiences	• All had received extensive training 1-1
		• All reported great support from home nurse and technician
		• Most reported making mistakes in the early weeks, and being scared or panicking
	Sources of learning	• Trial and error
		• Learning from the nurse or technician
		• From the manual
	Being exposed to different practices	• The initial practice as taught
		• Observing different nurses’ practices or attending a different dialysis unit
		• Practices change over time
Usability	Troubleshooting	• Real-time pressure display helps with troubleshooting
		• Some displays/alarms helpful for troubleshooting; others incomprehensible
	The challenges of remembering	• 14/19 reported forgetting to open/close all clamps
		• Several reported forgetting other details of the process
	Accessibility for the patient	• Screen and controls need to be easily accessed by patient, as well as carers
		• Display needs to be easy to read
		• Clamps need to be easy to use
	What don’t you like?	• The size and (lack of) portability
		• The time around dialysis getting things organized
Safety	Overview	• All participants considered it safe
		• All took care over infection control
		• Several had experienced untoward incidents
		• Most participants mentioned bubbles in the extracorporeal circuit
	Strategies for staying safe	• Avoid distractions; do not do when tired
		• Involve other people
		• Anticipate water or power problems
		• Give key to neighbour; keep mobile phone to hand
	Troubleshooting	• Various creative strategies were reported to enable dialysis to proceed when machine was not functioning properly
	Choosing quality of life	• People dialyse alone and when convenient, even if that reduces the available support
		• Some choose quality of life (time/location) over ‘best practice’

In presenting the results, direct quotations from participants are annotated to indicate type of participant (P = patient; C = carer; T = technician; N = nurse; D = nephrologist).

### Learning to use HHD technology

Learning to use the technology is an essential first step, and has a significant impact on both the quality of the patient experience and safety. HHD is at an extreme of complexity for a home self-care therapy. A patient, with or without a carer, is trained intensively at the dialysis unit for several weeks or months; for the participants in our study, this training was individualised. The training does not cover everything in detail, as there is simply too much information. All participants received home visits, particularly in the early weeks of dialysing at home, and both a specialist nurse and technician were available at the end of the phone, at least during working hours. When a patient first starts using their machine at home, they typically face teething issues and many reported making mistakes.

*I had one right at the beginning where it kept alarming and it, I couldn’t fathom out why. (P6)*

Most patients reported feeling scared or worried in the early months of HHD, particularly when something unfamiliar happened. Without prompting, 12 of the 19 patients/carers used the term ‘scared’ or ‘panic’ to describe feelings early on.

*We had no clue and it was really worrying. We were in a right old panic. (P14)*

They continue learning from their own experiences when encountering new situations not covered in training, such as dealing with unfamiliar machine alarms. Learning is often by trial and er.

*obviously you learn from your mistakes (C2)*

*It’s all by mistakes that you learn the machine, by the machine telling you. (P12)*

They also continue learning from interactions with the home therapies nurse, who they contact for machine-handling or patient-related issues, and from the renal technician, who they contact about more technical issues. For example, P10 had to be talked through how to take herself off the machine; she then noted down the procedure so as to be able to do it herself in future:

*And I phoned the unit up and she said take yourself off. […] one of the nurses talked me through it, and I said it’s not working; I said it’s not doing what you said. So she said, oh, I forgot to tell you, turn that off. So I turned it off, and then it worked as she said. So I wrote that down, because I thought if ever it happens again … (P10)*

Similarly, about 6 weeks in to HHD, P12 had to replace the bottle of disinfectant, and could not remember what he had been taught:

*I’d never changed one before. I was told how to do it. But you’d forgotten, you know what I mean? So, I rang up the technicians and he said, what does it say? And I read what it said on the machine and then he said, yes, your disinfectant’s empty at the back. I felt like a right [idiot]. (P12)*

Things gradually make more sense, and eventually the patient becomes an expert in using the technology. Participants drew analogies with following a recipe (P13) and with learning to drive (C11; P12). For example, C11 expressed it:

*when you start driving you’re very slow and… but after a few years driving, everything comes naturally and that’s the same with this. (C11)*

This sense of growing expertise generates confidence, reflected in their readiness to be flexible concerning session frequency and timing, including being prepared to dialyse at times when support from the dialysis unit is less comprehensive.

*During the day if I dialyse, I can phone the home unit, but if I have to phone the ward, and there’s a problem with this machine, they’re not very good on it. (P13)*

All participants reported relying on their nurses and technician as first ports of call in case of a problem. Most participants reported very positive experiences of particular nurses and technicians:

*There’s somebody at the end of the phone. The top one is [name]. She’s the Senior Sister. She’s like a mate of mine now. She’s good. (P12)*

*He’s great. [name]’s really good. Very funny. (P14)*

While most reported adhering strictly to the process as taught, a few reported being influenced by other practices they observe, whether due to differing practices in different units, by different members of staff, or as practices evolve:

*when I was at the other unit, [U1], I was there first, I found that the nurses there were very very strict when it came to the non-touch technique, you know, with hygiene. Then I found that in [U2] they were not so rigid. […] I always follow what you call it, the er [U1] unit (C2)*

*I certainly picked up a few things when we were in [holiday destination] that I could do a bit quicker (P14)*

*when you’re in the unit you find that all the nurses do it slightly differently. They all have their own little shortcuts and things (C3)*

*nowadays they do it in a different method, and [N1] sometimes has told me, oh, why don’t you try it like this, but it just confuses me. (P7)*

Two participants (P15, P16) explicitly suggested that their machines should be connected for remote monitoring. In the case of P16, he had connected his machine to the internet himself:

*the data that’s collected from the machine of any alarms, or, anything, and pump speeds, and all that, kind of, stuff gets sent straight to [supplier] in the USA. […] all I had to do was plug the Ethernet cable in. There’s a switch on the bottom that I had to switch, and then it just sits there, and does it itself. […] they’ve not allowed the technicians here to be able to collect that information.*

Technicians participating in this study also advocate remote monitoring and support. They reported two issues that make troubleshooting machine alarms over the phone difficult. Firstly, patients may have different terminologies for machine parts and secondly, technicians have to rely on their mental visualisation of what is happening. In some cases where the technician cannot ascertain the problem from the phone conversation, they have to ask the patient to come off the machine and lose the blood that is currently in the extracorporeal circuit.

### Usability of HHD technology

The five different machines in this study raised different issues; our aim is not to critique any particular system, but to draw out general findings across participants. Even when the initial learning curve is passed, the usability of the technology influences the quality and safety of the patient experience. It can improve patient satisfaction, reduce frustration, and lessen the likelihood of ambiguous situations that compromise safety. Many participants were very positive about the usability and safety of their HHD technology. We found that issues of usability arose particularly when troubleshooting problems and alarms during dialysis. Participants typically reported trying to solve problems directly with the machine first, then resorting to the manual, and finally calling a technician or nurse if needed. Some patients found using a manual helpful; others did not. Consulting a manual may not always be practical: for instance, a patient dialyzing on their own finds it difficult to manipulate a large manual with one hand.

*you don’t want to be opening the book every time an alarm goes off. (P13)*

*when an alarm would go off, and I didn’t understand, and I would try and solve it myself by looking through the booklet. But the booklet’s not really straightforward […] I couldn’t like, fully understand it. So I just called a technician to come and see it. (P4)*

The perception of staff was that patients did not refer much to the manual:

*If a patient is on the machine, he’s not gonna get the manual, sit there and read it. (T1)*

*The first thing a patient at home does is ring somebody; they don’t want to look in a book. (N1)*

Patients reported that they found some information on the technology’s interface useful in helping them deal with problems; for example, the real-time representations of pressures inside the dialysis lines help patients deal with arterial/venous pressure alarms. However, other machine alarms and messages are not understandable by the patient. The design is safe, as it does not allow the patient to proceed with treatment, but some of the machines in this study are not effective in supporting the patient in solving the problem. In one case, a carer reported having to dispose of blood because of a delay:

*if a few minutes pass by whilst you’re trying to sort this problem out, you then start to get stagnated blood which can begin to clot and on one occasion we had to actually lose the blood. (C19)*

As Piccoli et al. [22] note, “nothing is trivial in home hemodialysis”. Nearly all participants in this study reported having forgotten some aspect of the process. Most (14/19) had occasionally forgotten to open all clamps and one (P1) had forgotten to inject anticoagulant when needed.

Patients are advised to disconnect themselves from the machine within a few minutes of the end of the treatment. One patient (P9) programmed timers to alarm and wake him 45 and 30 minutes before the end. Another (P14) reported getting engrossed in other activities and not realising that her treatment was about to finish.

*sometimes I get so engrossed in what I’m doing we’ve finished and we’re not ready (P14)*

Many of the existing machines are designed for use by someone other than the patient. For patients who are dialysing alone, or who are trying to manage their own care with minimal intervention from others, such a design is inappropriate: they need access to the screen and controls while on dialysis and the screen to be legible, and they need clamps and controls to be usable.

*the screen is quite far away from me. This is the most eh…challenge, it’s not a challenge, but inconvenience, that the screen is…and in case of an alarm, if you are lying down it takes quite an effort to get to the screen, (P1)*

*I could do with the wording on the screen being bigger (P17)*

*the clamps…are too hard to clamp. […]it hurts the fingers and this is one thing but, because it’s hard to clamp when your blood pressure is low (P1)*

While there are clear arguments for delivering designs for HHD that are tailored towards the needs of home patients who are dialyzing independently, one participant (P13) highlighted an important consideration regarding support. This is that hospital nurses who do not specialize in supporting home care may be less able to provide remote support if the designs of the machines diverge to address the different needs of home and hospital patients.

*The people on the ward aren’t very familiar with this machine. […] Because they don’t have these machines on the ward (P13)*

When asked what they least liked about their systems, the most common response was its size. This related to two aspects. The first is the way that it tends to dominate within the home. E.g.:

*I’ve been invaded (C14)*

The second concerns its portability, in terms of allowing people to travel with their own dialysis equipment rather than either only ever going away for very short periods (typically one night) or booking in-centre dialysis while away. The latter can require considerable advance planning:

*It takes at least three months because you have to have Hep C, Hep B and HIV tests, blood work sent to that hospital. (P13)*

This highlights how significantly the patient experience is affected by the broader dialysis context, in terms of how care is managed across health service providers.

Another concern is the additional time (beyond direct dialysis time) that is needed for completing the dialysis process. This was noted by several participants.

While most participants reported adhering strictly to the way they were taught to dialyse, some reported developing short-cuts; for example,

*the regime was heat, disinfect, treatment, heat, disinfect, right? The engineers are saying and [manufacturer] are saying you don’t need to do that. Why heat, disinfect at the end when you’re going to heat, disinfect the next morning? So, what we do now is we do a rinse at the end, which is only nine minutes and not 40 minutes. (P15)*

### Safety during dialysis

Haemodialysis treatment requires many steps to be performed correctly and in the right order for treatment to be safe. Both patients and professionals generally perceived the machines as being safe:

*they are as safe as can be (T3)*

*the machine itself is, yes, it’s pretty idiot proof. (P16)*

One participant (P19) considers HHD to be safer than in-centre HD because of reduced risk of infection. Many participants explicitly commented on the importance of procedures to keep everything disinfected. E.g.:

*it’s important because, you know, it’s basically infection control. (P15)*

However, there are inherent risks of patient harm during dialysis, including: hypotension, accidental disconnection of dialysis needles or lines leading to blood loss, air embolism, clotting of blood in the extracorporeal circuit and haemolysis.

Patients were aware of these risks, and many reported having experienced one or more of these situations during their time on HHD. E.g.:

*I had a line disconnection, and there was blood everywhere […]it gets unscrewed. I’ve caught it a number of times unscrewed. (P16)*

*I felt very sick […] I fainted. (P19)*

*We had to come off and start again because there’s no way of clearing those bubbles. It was, like, really frothy in the drip chamber. (P17)*

Bubbles in the system were mentioned as a problem by 13/19 participants, though these were within the extracorporeal system, and bubbles were trapped before presenting immediate danger to the patient. Many participants reported having to ‘snap and tap’ for several minutes to get air out of the system, though one (P14) had resorted to simply waiting and allowing the air to clear itself:

*I’ve found that if I leave it another 15 minutes it gets a lot of the air out of the tubes. I don’t know how, why, or whatever, but it cuts down that snapping and tapping considerably. (P14)*

Some patients who were self-caring adopt strategies that minimise the likelihood of being distracted while preparing for dialysis, including talking everything through (P7), being alone (P10) and avoiding dialysing when already tired (P4).

*I literally talk to myself, you know, first you do this, then you do this, then you do this, and that way I know that I’m not going to make a mistake. (P7)*

*I like to do it on my own. I don’t like anyone here, because I have to think to do it (P10)*

*if I’m tired, I will just, I might just set up the machine, and then, so I’m fresh the next morning, and then just look at it and see that’s it’s set properly and then go on the machine. (P4)*

Conversely, to ensure their safety, many patients adopt strategies that involve other people during dialysis, for routine procedures and for troubleshooting; for example,

*every hour, [C12] comes up and she takes my blood pressure and writes down all these figures just to make sure. (P12)*

*I would never do it on my own. (P14)*

A home carer can engage in other activities while the patient is dialysing, and many carers in this study reported being elsewhere in the house during dialysis. Some had introduced a communication channel between the patient and themselves, e.g. walkie-talkies, to remain in touch. Two patients (P12, P17) relied on calling out for their carers in case of emergency, sometimes from a different floor of the house. Also, some patients dialyse when they are home alone, even if this is against the policy of their unit. In these cases, they rely on a neighbour or an untrained relative to provide assistance if needed. Some had given a neighbour a key to their home, and keep the neighbour’s number to hand to call in an emergency. Almost all participants in this study reported keeping a mobile phone at hand, with a list of numbers they might need to call (nurse, technician, neighbour and others). E.g.:

*I always have my telephone by my side. I told you, I get everything ready, all the telephone numbers I was given, water, electricity, whatever, technician, any emergency. (P5)*

Several participants articulated approaches to anticipating problems and avoiding them. For example, just as P4 reported delaying dialysis if she was tired, so several participants reported anticipating water usage in the home and P13 reported anticipating possible problems with the power:

*If it’s really windy it blows the power out, so I tend not to go on if it’s really stormy outside. (P13)*

No safety issues were identified for the machines involved in this study, when used as designed, for issues within the scope of what the technology can detect. The machines are effective in ensuring that all required steps are performed before letting the patient proceed to treatment. This gives patients confidence in doing their treatment independently. In fact, some patients rely on the ‘safety-consciousness’ of the machine during interactions in the early stages of learning: they make mistakes, and learn through these, knowing that the machine would not let them proceed to treatment if they missed a step. This allows them to gradually learn how to perform a complex treatment.

*There’s a lot of safety features built in, and if you don’t do everything in the set order, the machine will tell you. (P3)*

Some steps in a dialysis session are outside the scope of what current HHD technology can detect, so the technology cannot ensure that the patient does them correctly. For example, when the patient performs the disconnection and reconnection procedure at the end of treatment to rinse the blood left in the circuit back into their body, the machine cannot check whether the patient connected the ends correctly. One patient in our study (P19) reported having incorrectly connected the two ends of the dialyser once, but her carer spotted the error before it caused difficulties.

Some participants had attempted to fix machine problems themselves, instead of waiting for the technician to visit or arranging to dialyse in-centre. One carer (C2) fixed a water leak behind the machine with tape and proceeded to dialyse his father, even though the technician had told him not to use the machine until he had come to fix the leak. Another patient (P13) used a hair dryer to evaporate some water inside his machine that was preventing it preparing the dialysate. These could be seen as deliberately taking a safety risk, though a crucial factor in both these scenarios was the desire of a sick patient to dialyse as soon as possible to feel better. There is also a wider context: when a home patient’s machine is faulty, some participants have experienced difficulties arranging in-centre dialysis, due to limited capacity.

*if you phone [the unit], the… very likely they don’t have beds. And the slot that he probably wants, early in the morning, they probably won’t have it; no afternoon slot, possibly evening slot. And if you go to the evening slot, it’s five o’clock until 11 o’clock, and that’s too stressful for [P2]. (C2)*

This is a further example of how patients’ and families’ perceptions of the broader care system influence their decisions about care management, and hence of how they stay safe while also achieving effective treatment.

One participant recounted how he had ‘tricked’ the machine to enable him to come off in a controlled way:

*I’d started dialysis, and the batch was going to run out, and it ran out, but I couldn’t set the machine to come off. It had alarmed, I’d done all the stuff to cancel the alarm, but because it couldn’t reset itself it then kept alarming. So, what I did was, I thought, at the beginning this is all full of saline, and not full of dialysate. I thought, why don’t I just put saline in it, and trick it into thinking that the batch is still there and made up? So, what I did was I got a 60 mil syringe, filled it with saline, and connected it to the dialysate line. So, then when it tried to reset its alarms and everything it was just drawing in saline from the syringe, so that I could then sort it all out to come off. […] I managed to sort it out so that I could come off, otherwise I would have lost a whole circuit of blood.[…] I also wouldn’t have got any information from the machine as to how long I did, how many litres of dialysate I’d actually done. (P16)*

Other participants reported taking actions that improve their quality of life while possibly compromising safety. For example, C2 speeds up the machine at the beginning and end of dialysis so that the patient can get through the process faster:

*The wash-back… increases, you know, just to get it… yes, just to make things easier for [P2], you know. Because, you know, at the end he’s very, very tired and he wants to get off as quickly as possible (C2)*

Taking a different focus on comfort, P8 dialyses on her verandah, even though that requires her to have a patio heater to keep the dialysate at the required temperature. These are examples of patients thinking beyond ‘best practice’ of dialysis to achieve broader goals of personal wellbeing.

## Discussion

These findings about how patients and carers learn how to dialyse at home, use the HHD technology, and stay safe highlight areas for potential improvement in the design and delivery of HHD, including the design of the machine, channels of communication with specialist nurses and technicians, and the broader socio-technical system. In this section, we draw out design requirements that have been highlighted through this study. These requirements are summarised in Table 3. Some of these are addressed by current technologies; others require further developments. There is no single solution that is optimal for all; for example, size of machine and quality of dialysis may need to be traded off against each other.

**Table 3:** **Summary of areas for improvement (design of machines and surrounding care provision)**

**Enhancing learning, safety and usability**	• Better clamping mechanism (easier to do, easier to remember)
	• Better support for learning, troubleshooting and remembering all steps
	• Providing prompts to alert the patient when dialysis is about to finish
	• Designing interfaces for use by patient *or* carer
	• Easy intervention by untrained person in emergency
	• Minimize risk of incorrect connections (through colour coding or connector types)
	• Provide guidance on troubleshooting out-of-hours for home machines
	• Streamlining the extra processes before and after dialysis
	• Providing reminders for periodic activities (e.g. changing filters, making up batch of dialysate)
	• Provide access to in-centre dialysis at short notice
**Remote monitoring**	• Real-time remote monitoring of current machine state advocated by technicians
	• Remote intervention in case of emergency
	• Nephrologist wanted to be able to send messages to patients
	• Patients wanted to be able to send readings to clinic

Some issues relate to the learnability and usability of the machine. The interface design of HHD technology can contribute to a smooth learning experience for the patient. For example, one machine in this study provided contextual information to the patient and walked the patient through step by step for many tasks. There is no need to overload the patient with information during the training: they can continue learning at home through the technology. This also helps them to deal with situations that happen very rarely, which may have been learnt but then forgotten.

Where possible, the technology should display possible causes and solutions, instead of just stating that there is a problem. The patient experience could also be improved by having messages that do not contain technical terms and are simpler to understand. One of the machines that featured in this study had an alarm indicator that showed whether to call a nurse or a technician for guidance on how to solve the problem, which patients found helpful.

As well as walking the patient through, the technology could be designed to help patients and carers coordinate phases of treatment and remind them of steps to be performed. This includes not just steps with the machine but also steps for phases of treatment that they need to coordinate themselves. An example would be having a physical placeholder for organising items that are needed during dialysis. As noted above, the problem most frequently reported by participants was forgetting to open (or close) all clamps, so a way of simplifying this process would probably be the greatest ‘win’ from a patient’s perspective. Providing reminders might extend beyond a single treatment to activities that need to be performed periodically; for example, some systems prompt the patient when the filter in the machine needs changing, or when disinfection should be carried out. This avoids patients having to keep track of these requirements themselves.

As noted above, patients need time to prepare for coming off the machine. The machine could provide cues to inform the patient that treatment will finish soon; this would facilitate preparing for disconnection, to minimise risks of clotting and haemolysis.

Haemodialysis is safety-critical. The design of HHD technology should allow untrained people to intervene in case of emergency. This approach is exemplified by one machine in our study, which allows a helper with little or no training to intervene in a hypotensive episode: a single button press is required, which both suspends fluid removal and dispenses fluid to the patient.

Another area of vulnerability identified in this study is in making connections. It is important to mitigate the risk of incorrect connections, e.g. making colour-coding very clear so that patients can easily distinguish between the two lines: a suggestion also made by Allcock et al. [7]; an alternative might be to introduce different kinds of connectors for the different lines so that it is physically impossible to make the wrong connections.

For patients who self-care, easy access to displays and controls are essential. Greatest flexibility (to support both self-care and care by someone else) is provided by configurable controls – e.g., a separate networked control panel, with adjustable display.

Although they may dialyse at home, patients are not alone, and often rely on support from nurses, technicians and family. One improvement that could help learning and communication would be the use of remote monitoring to provide a direct channel between the patient’s machine and clinicians and technicians. Remote monitoring would increase the chance of the technician understanding the problem, and hence being able to provide optimal support. Cafazzo et al. [11] found that, for patients transitioning to nocturnal HHD, remote monitoring helped patients cope with teething issues. This applies also for patients transitioning from in-centre haemodialysis to HHD. This could extend beyond support to intervention if needed. For example, the measures for dealing with hypotension could be triggered remotely by monitoring staff, or monitoring staff could call for an ambulance if they detect that something is wrong and do not get a response from the patient, as discussed by Schlaeper and Diaz-Buxo [9]. As well as remote monitoring, D1 highlighted the possibility of clinicians sending messages to the patient via the system and two patients proposed that the system should be able to automatically record and upload information required by clinicians for tracking their progress over time.

One of the challenges noted above is that some of the requirements for home use (where the machine is a ‘personal technology’) are different from those of a machine to be used in hospitals (where clinical staff are on hand and the machine is used for many patients). Where machines are tailored to home use, it is nevertheless important that patients can receive remote support 24/7, so there is a need to provide out-of-hours nursing support for all machines. This might be coordinated across healthcare providers rather than being localised to a particular provider to achieve economies of scale.

Like any complex technology, HHD machines occasionally develop faults, and people sometimes resorted to ‘quick fixes’ to make dialysis possible. It should be possible for HHD patients to access in-centre dialysis at short notice.

The patient is not alone: their place in a broader socio-technical system should be recognized and addressed. Patients’ interactions with the technology can be influenced by variations in practices across clinicians and renal units: clinicians from other renal units can have dialysis procedures which differ from those in the patient’s local unit; the perspectives may even conflict. While some patients stick strictly to the specific steps learnt during their training, others adapt procedures as they become aware of alternatives. This underlines the importance of supporting patients in building knowledge and facilitating their access to information on HHD, so that they can make informed decisions on how to use the technology.

### Limitations

This study involved 19 patients (and their carers) under the care of four hospitals in the UK, and all participants consented freely to take part. Consequently, their views and experiences represent those of ‘successful patients’. Further, many of them regard being able to dialyse at home as a privilege, so although they were comfortable discussing past difficulties, they may have been reluctant to discuss ongoing difficulties for fear of having their competence questioned.

The data reflects participants’ current experiences, and does not anticipate new developments. T3 notes:

*There are new dialysis machines coming through at the moment from certain companies that you can actually tap into. So you can actually go online, call up its IP address and actually go into the actual machine to see what the machine is doing. (T3)*

Such developments are already addressing some of the requirements identified with participants in this study. In these cases, the study confirms the value of such developments for the future of HHD.

Finally, there are factors in the delivery of HHD that are outside the scope of this report; for example, interactions with other patients currently play only a small role in patients’ experiences and have therefore been omitted from the analysis and discussion presented here, and D1, the only nephrologist interviewed, highlighted set-up cost as a significant challenge in the provision of HHD.

## Conclusions

The focus of this study has been on how the design of HHD technology and the broader care context can facilitate the learning process, support the patient, and ensure safety during dialysis. As others (e.g. [16, 23, 24]) have noted, and as several participants reported, patients are people who are trying to live their lives as best they can. A minority of patients seemingly walk a thin line between ensuring safety during dialysis and improving their overall experience of using the technology. Both the technology and the training on how to use it should be designed to help people achieve the best possible balance between safety and quality of life. This stresses the need for supporting patients in developing “their own authority” [25].

There may not be a uniquely ideal design solution: a system that is compact enough to be portable for one patient may not offer the quality of dialysis needed by another; a user interface and controls that are easily accessed by the patient who is self-caring may not be suitably placed for a carer or nurse; a machine that is tailored for home use may be so different from one tailored to the demands of hospital or dialysis centre use that staff familiar with one may be unable to guide patients using the other. As well as highlighting the strengths and limitations of current generation machines for manufacturers, our intention is that the design considerations presented above should inform clinicians, those responsible for procurement, and those involved in the provision and commissioning of HHD services.

If home dialysis is to be more widely taken up, there is a need to better understand the needs of patients, both cognitive and affective, as they transition from hospital to home care: giving people the support and confidence in setting up and troubleshooting, particularly in the early weeks of home use. One technician in this study asserted that *“unit patients live to dialyse, home patients dialyse to live”* (T1), and another described HHD’s effect as “*it brings their life back*” (T3): these strong statements recognise the importance of quality of life for home patients. Patient safety and patients’ and their families’ quality of life are enhanced by designing machines to fit in the home environment and to be as efficient as possible to set up and use, and enabling people to understand the potential consequences of the decisions that they make when adapting procedures and when troubleshooting. In this paper, we have highlighted some potential approaches to improving patient safety and experience on HHD.

## Abbreviations

HHD: Home haemodialysis.
